# Imported Cutaneous Diphtheria, Germany, 1997–2003

**DOI:** 10.3201/eid1102.040560

**Published:** 2005-02

**Authors:** Andreas Sing, Jürgen Heesemann

**Affiliations:** * Max von Pettenkofer-Institut für Hygiene und Medizinische Mikrobiologie, Munich, Germany

**Keywords:** letter, diphtheria, Corynebacterium, cutaneous diptheria

**To the Editor:** The March 2004 report by de Benoist et al. on the incidence of imported cutaneous diphtheria in the United Kingdom (*1*) prompted us to describe the situation of cutaneous diphtheria in Germany and to analyze the cases reported to the German Consiliary Laboratory on Diphtheria since its establishment at our institute in 1997. The laboratory provides advisory and diagnostic services mainly to microbiologic laboratories throughout Germany.

From 1997 to 2003, 6 cases of cutaneous infections caused by toxigenic *Corynebacterium diphtheriae* were documented (Table). None of these was accompanied by secondary diphtheria infection. Toxigenicity was determined by both *dtx* polymerase chain reaction and Elek test (*2*). As in the United Kingdom, all cases for which clinical information was available (N = 5) were imported. Three were found in tourists who had traveled to tropical countries: a 20-year-old diver had injured her heel after stepping on coral in Thailand; a 60-year-old tourist had a chronic ulcer in the thigh after a trip to Indonesia (no history of an insect bite); and a 39-year-old traveler to Kenya returned with a purulent ear infection with no memory of trauma or insect bite. The remaining imported *C. diphtheriae* skin infections were reported in 2 Angolan children, 5 and 10 years of age, who were brought to Germany by a humanitarian organization for surgery on severe gun wounds to their lower extremities (foot and thigh with chronic osteomyelitis, respectively). To our knowledge, these reports are the first of cutaneous diphtheria in gunshot wounds in recent years. Moreover, in the patient with the thigh wound, *C. diphtheriae* was also isolated from a deep fistula, which suggests involvement of *C. diphtheriae* in the chronic osteomyelitis.

**Table:** Cutaneous diphtheria cases caused by toxigenic *Corynbacterium diphtheriae* in Germany, reported to the German Consiliary Laboratory, 1997–2003

Y	Sex*	Age (y)	Biotype	Localization	Country	Vaccination status
1997	F	20	*mitis*	Heel	Thailand	+†
2000	M	60	*mitis*	Thigh	Indonesia	+†
2001	M	60	*mitis*	NR‡	NR‡	NR‡
2001	F	39	*mitis*	Ear	Kenya	(+)§
2002	M	5	*mitis*	Foot; gun wound	Angola	–¶
2002	M	10	*mitis*	Thigh; gun wound	Angola	–¶

*M, male; F, female. † +, vaccination status as recommended in Germany (basic and booster immunization). ‡ NR, not reported. § (+), vaccination status only partial as recommended in Germany (only basic immunization). ¶ –, not vaccinated.

As in the United Kingdom, all cases of diphtheria reported since 1997 were caused by *C. diphtheriae mitis*. In 4 of 5 cutaneous diphtheria patients who had an available medical history, mixed infections with *Staphylococcus aureus* and *Streptococcus pyogenes* were found; 3 of 5 patients were not sufficiently vaccinated against diphtheria as recommended. Systemic symptoms, such as malaise and general weakness, developed in the 20-year-old Thailand tourist, although she had received a booster dose just before her travel. Cutaneous diphtheria must be expected even in vaccinated patients; for instance, among serum samples of 287 healthy German adults with a complete record of basic immunization against diphtheria, only 42.2% showed full serologic protection as indicated by antitoxin levels >0.1 IU/mL (*3*).

As de Benoist et al. outline, cutaneous diphtheria might be difficult to diagnose because of its unspecific clinical appearance and the presence of mixed infections in chronic nonhealing skin lesions. Because of the nearly complete disappearance of cutaneous diphtheria in many parts of the western world, microbiologists lack experience in identifying *C. diphtheriae* grown from specimens. From 1997 to 2003, approximately one fifth of the strains sent to our Consiliary Laboratory on Diphtheria for species identification and toxin testing were either nondiphtheria *Corynebacterium* spp. or noncoryneform bacteria of different genera (including lactobacilli, *Dermabacter hominis*, and *Propionibacterium acnes*).

Clinicians (*4*) and microbiologists (*5*) should be aware of the possibility of cutaneous diphtheria in chronically infected skin lesions in patients returning from disease-endemic regions. Medical personnel should include this in civilian as well as military health services, since our cases indicate that toxigenic *C. diphtheriae* might affect not only travel-related skin injuries caused by leisure or tourist activities but also wounds in patients from war regions in diphtheria-endemic areas.
